# Actin Cytoskeletal Reorganization Function of JRAB/MICAL-L2 Is Fine-tuned by Intramolecular Interaction between First LIM Zinc Finger and C-terminal Coiled-coil Domains

**DOI:** 10.1038/s41598-019-49232-8

**Published:** 2019-09-05

**Authors:** Kazuhisa Miyake, Ayuko Sakane, Yuko Tsuchiya, Ikuko Sagawa, Yoko Tomida, Jiro Kasahara, Issei Imoto, Shio Watanabe, Daisuke Higo, Kenji Mizuguchi, Takuya Sasaki

**Affiliations:** 1 Department of Biochemistry, Tokushima University Graduate School of Medical Sciences, Tokushima, 770-8503 Japan; 2 Department of Interdisciplinary Researches for Medicine and Photonics, Institute of Post-LED Photonics, Tokushima University, Tokushima, 770-8506 Japan; 3 Intelligent Bioinformatics Research Team, Artificial Intelligence Research Center, The National Institute of Advanced Industrial Science and Technology, Tokyo, 135-0064 Japan; 4 National Institutes of Biomedical Innovation, Health and Nutrition, Ibaraki, 567-0085 Japan; 5 Support Center for Advanced Medical Sciences, Tokushima University Graduate School of Biomedical Sciences, Tokushima, 770-8503 Japan; 6 Department of Neurobiology and Therapeutics, Faculty of Pharmaceutical Sciences, Tokushima University, Tokushima, 770-8503 Japan; 7 Division of Molecular Genetics, Aichi Cancer Center Research Institute, Nagoya, 464-8681 Japan; 8 Department of Cancer Genetics, Nagoya University Graduate School of Medicine, Nagoya, 466-8550 Japan; 9 Thermo Fisher Scientific, Chromatography & MS Department, Application Group, LC-MS, Yokohama, 221-0022 Japan

**Keywords:** Molecular modelling, Actin, GTP-binding protein regulators

## Abstract

JRAB/MICAL-L2 is an effector protein of Rab13, a member of the Rab family of small GTPase. JRAB/MICAL-L2 consists of a calponin homology domain, a LIM domain, and a coiled-coil domain. JRAB/MICAL-L2 engages in intramolecular interaction between the N-terminal LIM domain and the C-terminal coiled-coil domain, and changes its conformation from closed to open under the effect of Rab13. Open-form JRAB/MICAL-L2 induces the formation of peripheral ruffles via an interaction between its calponin homology domain and filamin. Here, we report that the LIM domain, independent of the C-terminus, is also necessary for the function of open-form JRAB/MICAL-L2. In mechanistic terms, two zinc finger domains within the LIM domain bind the first and second molecules of actin at the minus end, potentially inhibiting the depolymerization of actin filaments (F-actin). The first zinc finger domain also contributes to the intramolecular interaction of JRAB/MICAL-L2. Moreover, the residues of the first zinc finger domain that are responsible for the intramolecular interaction are also involved in the association with F-actin. Together, our findings show that the function of open-form JRAB/MICAL-L2 mediated by the LIM domain is fine-tuned by the intramolecular interaction between the first zinc finger domain and the C-terminal domain.

## Introduction

In our previous studies, junctional Rab13-binding protein (JRAB)/molecule interacting with CasL-like2 (MICAL-L2) is identified as an effector protein of Rab13^1^, a member of the Rab family of small GTPases (Rab), which contributes to the regulation of membrane trafficking^2–4^. We also showed that Rab13-JRAB/MICAL-L2 is involved in the transport of cell adhesion molecules and the formation of cell–cell adhesion in epithelial cells^1,5,6^. In addition, JRAB/MICAL-L2 regulates actin cytoskeletal reorganization during epithelial junctional development^6,7^. JRAB/MICAL-L2 consists of the N-terminal calponin homology (CH) domain, LIM domain, and C-terminal coiled-coil (CC) domain linked by intrinsically disordered region. The LIM domain and the latter part of disordered region bind to actin filaments (F-actin), followed by the stabilization and the bundling of F-actin, respectively^7^. From these, JRAB/MICAL-L2 exhibits multiple actin cytoskeletal reorganization through a certain regulatory mechanism, e.g. the protein conformational change. Indeed, we generated a structural model of JRAB/MICAL-L2 through a combination of biochemical analyses and bioinformatics, and then presented evidence for a conformational change of JRAB/MICAL-L2 (between open and closed form) upon association with Rab13^8^. In the structure model, JRAB/MICAL-L2 adopts a closed form via the intramolecular interaction between the N-terminal LIM domain and the C-terminal CC domain. Rab13 competes with the LIM domain for a part of JRAB/MICAL-L2 C-terminus, leading to the conformational change of JRAB/MICAL-L2 from closed to open form. Recently, our interdisciplinary approach proved that JRAB/MICAL-L2 regulates collective cell migration through the spatiotemporal regulation of actin cytoskeleton depending on the conformation^8,9^. Closed-form JRAB/MICAL-L2 forms thick F-actin bundles along the leading edge, followed by generation of traction force that pulls the cell population^7,8^. On the other hand, open-form JRAB/MICAL-L2 forms ruffles without thick F-actin bundles at the free border, leading to release from the traction force; it mainly localizes at cell–cell contacts, probably for reinforcement of the cell–cell adhesions^8^. Moreover, we found that the well-known actin-binding proteins, filaminA and actinin-1/-4, preferentially associate with open-form JRAB/MICAL-L2^7,10^. In epithelial cells, actinin-4 recruits JRAB/MICAL-L2 to the cell-cell contacts where open-form JRAB/MICAL-L2 functions^11^. In fibroblasts as well as epithelial cells, JRAB/MICAL-L2 induces various phenotypes depending on its conformation. Closed-form JRAB/MICAL-L2 enhances the formation of stress fibers, whereas open-form JRAB/MICAL-L2 induces the formation of peripheral ruffles via an interaction between its CH domain and filamin^10^. To understand how JRAB/MICAL-L2 performs its various functions, it is necessary to establish a linkage between its phenotypes and conformations. To this end, we focused on the LIM domain of JRAB/MICAL-L2, for the following reasons. First, the N-terminal LIM domain of JRAB/MICAL-L2 binds to the C-terminal CC domain, yielding the closed conformation^7^. Second, the LIM domain associates with F-actin and then inhibits F-actin depolymerization^7^. In this study, we found that the LIM domain, independent of the C-terminus, is also necessary for the function of open-form JRAB/MICAL-L2. In mechanistic terms, two zinc finger domains within the LIM domain bind the first and second molecules of actin at the minus end, potentially inhibiting the depolymerization of F-actin. The first zinc finger domain also contributes to the intramolecular interaction of JRAB/MICAL-L2. Moreover, the residues of the first zinc finger domain that are responsible for the intramolecular interaction are also involved in the association with F-actin. Together, we concluded that the function of open-form JRAB/MICAL-L2 mediated by the LIM domain is fine-tuned by the intramolecular interaction between the first zinc finger domain and the C-terminal domain.

## Results

### HDX-MS analyses reveal the intramolecular interaction site in the LIM domain of JRAB/MICAL-L2

**Figure 1:**
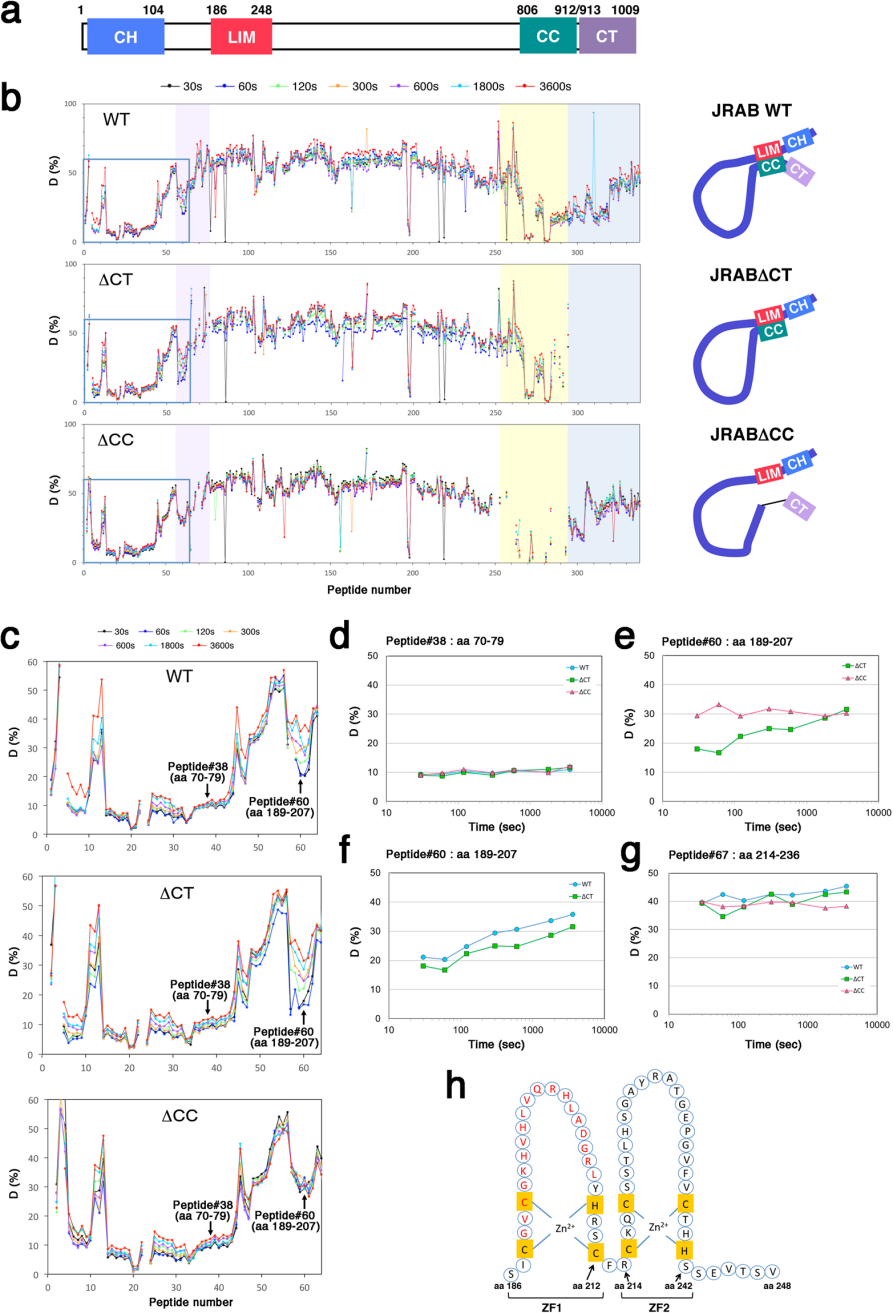
Application of hydrogen/deuterium exchange mass spectrometry (HDX-MS) to determination of the intramolecular interaction site of JRAB/MICAL-L2. (**a**) Schematic indicates the structure of mouse JRAB/MICAL-L2. Numbers represent amino acid positions. (**b**) HDX-MS analyses of JRAB WT and variants. Each recombinant protein, exchanged by timed exposure to the D_2_O buffer, was dissociated with chemical denaturants, and then passed through immobilized pepsin column. The proteolytic peptides were roughly resolved by liquid chromatography–mass spectrometry (LC-MS) in order to measure the deuterium incorporation into each peptide. WT: JRAB WT, ΔCT: JRABΔCT, ΔCC: JRABΔCC. Line color corresponds to the indicated incubation time on the top (30, 60, 120, 300, 600, 1800, or 3600 s). The area colored in violet indicates the LIM domain, yellow indicates the CC domain, and dark blue indicates the CT domain. Schematic indicates the putative conformation of JRAB WT and variants (right). (**c**) Enlarged view of light-blue square (peptide #1–64) in (**b**). Arrows indicated the positions of peptide #38 (aa 70–79) and peptide #60 (aa 189–207). (**d**–**g**) Time course of the deuterium incorporation into the indicated peptide of JRAB WT or variants. (**d**) peptide #38 (aa 70–79; EEQLGIPALL); (e)(f) peptide #60 (aa 189–207; GVCGKHVHLVQRHLADGRL); (**g**) peptide #67 (aa 214–236; RCKQCSSTLHSGAYRATGEPGVF). JRABΔCT and JRAB WT showed the slower exchange rates than JRABΔCC in peptide #60. (**h**) Schematic indicates amino acid residues of the LIM domain of JRAB/MICAL-L2. The LIM domain consists of two tandem-arranged zinc fingers (ZF1; aa 186–212, ZF2; aa 214–242). Red indicates the putative JRAB-C binding residues identified by HDX-MS analyses.

JRAB/MICAL-L2 consists of the N-terminal CH domain, LIM domain, and the C-terminal CC domain linked by intrinsically disordered region (Fig. 1a). Previously, we performed biochemical analyses using truncated recombinant proteins to show that the N-terminal LIM domain interacted with the C-terminal CC domain (amino acids [aa] 806–912), leading to the intramolecular interaction, and that this interaction was disrupted competitively by the binding of Rab13 to the C-terminal region (aa 877–1009) including CT domain (aa 913–1009)^8,12^. By a combination of bioinformatic and biochemical analyses, we then generated a structural model that suggested that the hydrophobic or negatively charged region of the C-terminal part of JRAB/MICAL-L2 is involved in the interaction with the deduced structure of the N-terminal LIM domain and Rab13^8^. We also developed two deletion mutants of JRAB/MICAL-L2: JRABΔCT, which lacks the CT domain, and JRABΔCC, which lacks the CC domain (Supplementary Fig. S1), and showed that JRABΔCT and JRABΔCC adopt the constitutively closed and open forms, respectively^8,12^. In this study, we tried to narrow down the exact sites responsible for the intramolecular interaction, in order to obtain new insight into the relationship between the conformational dynamics of JRAB/MICAL-L2 and its function. For this purpose, we utilized hydrogen/deuterium exchange mass spectrometry (HDX-MS), a key technique for monitoring structural and dynamic aspects of proteins in solution^13,14^. We expressed hexahistidine (His)-tagged wild-type JRAB (JRAB WT) or the two mutants in HEK293 cells, and then purified each recombinant protein using immobilized-metal affinity chromatography, followed by gel filtration column chromatography as previously described^8^. After each protein (3 μg) was diluted in deuterium oxide (D_2_O) buffer and incubated for a certain period of time (30, 60, 120, 300, 600, 1800, or 3600 sec) at 10 °C, protein labeling was quenched by addition of guanidine HCl (low pH), and then the labeled protein was placed at 0 °C to decrease the exchange rate. Individual labeled protein at different incubation period was digested with pepsin, and then subjected to liquid chromatography–mass spectrometry (LC-MS) to measure the deuterium incorporation in each peptide. When the peptide is free from intramolecular interaction, it reaches maximum deuterium uptake at the shortest incubation time. That is because there is nothing to disturb the deuterium incorporation in the peptide. On the other hand, the peptide masked by intramolecular interaction needs longer incubation (up to 3600 sec) until it reaches maximum deuterium uptake. Most peptides among His-JRAB WT, His-JRABΔCC, and His-JRABΔCT were exchanged rapidly (Fig. 1b); for example, peptide #38 (aa 70–79) of the three proteins reached maximum deuterium uptake within 30 sec (Fig. 1c,d). However, in peptide #60 (aa 189–207) of the first zinc finger (ZF1: aa 186–212) within the LIM domain, His-JRABΔCT showed a slower exchange rate than His-JRABΔCC (Fig. 1c,e), but no difference between His-JRAB WT and His-JRABΔCT was observed (Fig. 1c,f). By contrast, in both mutants and JRAB WT, peptide #67 (aa 214–236) in the second ZF (ZF2: aa 214–242) was exchanged rapidly within 30 sec (Fig. 1g). As for the C-terminal CC region, the corresponding region of His-JRAB WT and JRABΔCT was barely exchanged after 1 h (Fig. 1b). Together, the peptide #60 (aa 189–207) in ZF1 of JRABΔCT and JRAB WT displayed the slower exchange rates than that of JRABΔCC. From the difference of exchange rate between His-JRABΔCT and His-JRABΔCC, the region of ZF1 (aa 189–207) represents the binding domain against the C-terminus (Fig. 1h, schema).

### The first zinc finger in the LIM domain is necessary for the intramolecular interaction of JRAB/MICAL-L2

**Figure 2:**
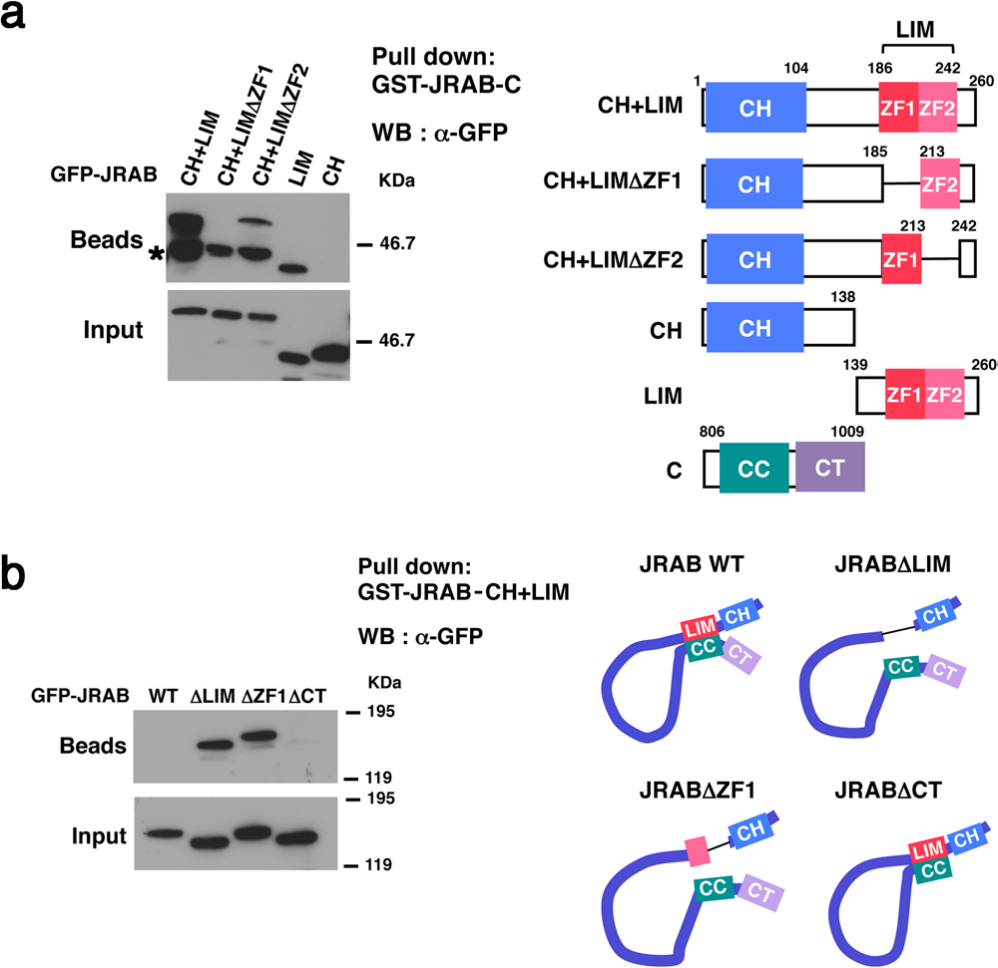
The first zinc finger of JRAB-LIM is essential for the interaction with JRAB-C. (**a**) HEK293 cell lysates containing GFP-tagged JRAB variants (CH + LIM, CH + LIM∆ZF1, CH + LIM∆ZF2, LIM, or CH) were subjected to pull-down assays using GST-JRAB-C. The pulled-down protein was detected by western blotting (WB) using anti-GFP antibody (left). Asterisk, nonspecific bands. Schematic indicates the structure of JRAB variants. Numbers represent amino acid positions. (right). (**b**) HEK293 cell lysates containing GFP-tagged JRAB variants (WT, ∆LIM, ∆ZF1, ∆CT) were subjected to pull-down assays using GST-JRAB-CH + LIM. The pulled-down protein was detected by WB using anti-GFP antibody (left). Schematic indicates the conformation of JRAB variants (right). (**a**,**b**) Full-length blots are presented in Supplementary Fig. S6.

To determine whether ZF1 (aa 186–212) of JRAB/MICAL-L2 is responsible for the interaction with the C-terminal CC domain, we prepared plasmids encoding GFP-JRAB-CH + LIMΔZF1 (aa 1–185 + 213–260), lacking ZF1, and GFP-JRAB-CH + LIMΔZF2 (aa 1–213 + 242–260), lacking ZF2, and then performed pull-down assays with GST-JRAB-C (aa 806–1009), which contains the C-terminal CC domain. GFP-JRAB-CH + LIM (aa 1–260) and GFP-JRAB-LIM (aa 139–260) bound to GST-JRAB-C as previously described^12^, and GFP-JRAB-CH + LIMΔZF2 also bound to it (Fig. 2a). However, as with the negative control GFP-JRAB-CH (aa 1–138), GFP-JRAB-CH + LIMΔZF1 did not bind to GST-JRAB-C.

Next, we prepared one more mutant to show that ZF1 is the specific region involved in the intramolecular interaction within JRAB/MICAL-L2: GFP-JRABΔZF1, which lacks the ZF1 region (aa 186–212) (Supplementary Fig. S1). We then performed pull-down assays to determine whether the mutant assumes the open or closed form. Previously, we used GST-JRAB-C for pull-down assays to detect JRAB-N free of its own C-terminus, but this time we used GST-JRAB-CH + LIM because GFP-JRABΔZF1 might not contain the binding region for the C-terminal CC domain. As expected, GFP-JRABΔCT, which assumes the constitutively closed form, did not bind to GST-JRAB-CH + LIM (Fig. 2b). By contrast, GFP-JRABΔZF1 bound to GST-JRAB-CH + LIM, indicating that the mutant remained in the open form. GFP-JRABΔLIM, which lacks the region including LIM domain (aa 139–260) (Supplementary Fig. S1) was used as a positive control in these experiments. GFP-JRAB WT was pulled down by GST-JRAB-C, but to a lesser extent than by GFP-JRABΔCC^8^. However, GFP-JRAB WT was not pulled down in an assay using GST-JRAB-CH + LIM. This result suggests that pull-down assays using GST-JRAB-CH + LIM are less sensitive than the assays using GST-JRAB-C for detection of the intramolecular interaction of JRAB/MICAL-L2.

These results support the idea that JRAB/MICAL-L2 changes its conformation through an intramolecular interaction between the N-terminal ZF1 in the LIM domain and the C-terminal CC domain.

### Prediction of binding sites by docking simulation based on the result from HDX-MS reveals which residues contribute to the intramolecular interaction of JRAB/MICAL-L2

**Figure 3:**
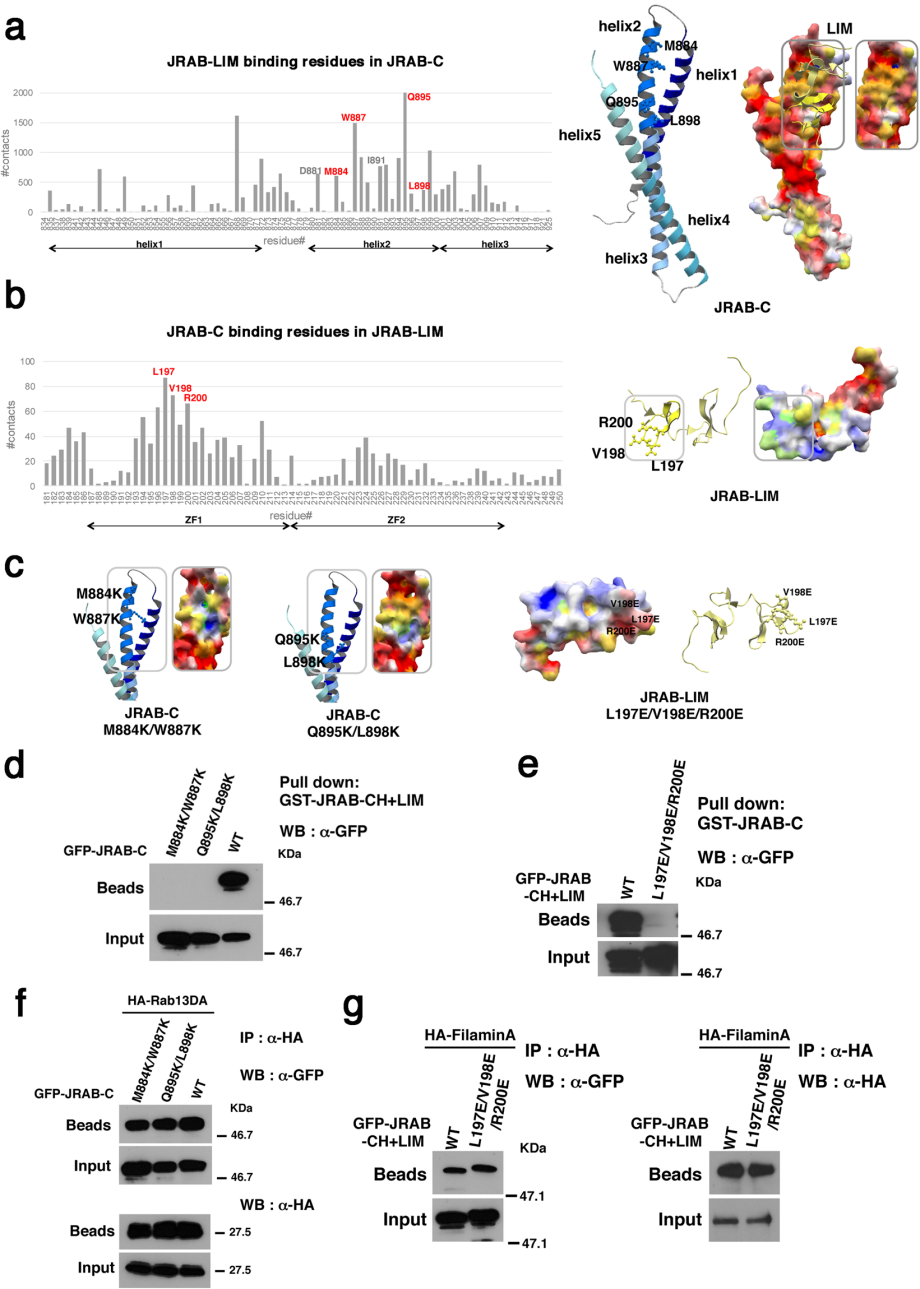
Prediction of binding sites by docking simulation based on the result from HDX-MS reveals which residues contribute to the intramolecular interaction of JRAB/MICAL-L2. (**a**,**b**) Numbers of contacts of residues in JRAB-C with JRAB-LIM (a), and in JRAB-LIM with JRAB-C (**b**), in the docking poses between the structural models of JRAB-C and JRAB-LIM. The residues colored in red that appeared frequently, M884, W887, Q895, and L898 in helix 2 of JRAB-C (**a**) and, L197, V198, and R200 in JRAB-ZF1 (b), were selected as candidates for the residues involved in the interaction between JRAB-C and JRAB-LIM. Electrostatically positive and negative regions on molecular surfaces are depicted in blue and red, respectively, and hydrophobic residues in yellow. (**c**) Structures of two JRAB-C double mutants (M884K/W887K and Q895K/L898K) and JRAB-LIM triple mutant (L197E/V198E/R200E), in which the side-chain conformations of the mutated residues were predicted using Scwrl 4.0. (**d**) HEK293 cell lysates containing GFP-tagged JRAB-C WT or mutant (M884K/W887K or Q895K/L898K) were subjected to pull-down assays using GST-JRAB-CH + LIM. The pulled-down protein was detected by WB using an anti-GFP antibody. (**e**) HEK293 cell lysates containing GFP-tagged JRAB-CH + LIM WT or mutant (L197E/V198E/R200E) were subjected to pull-down assays using GST-JRAB-C. The pulled-down protein was detected by WB using anti-GFP antibody. (**f**) Cell lysates from HEK293 cells expressing GFP-JRAB-C WT or mutant (M884K/W887K or Q895K/L898K) together with HA‐Rab13DA were immunoprecipitated (IP) with anti‐HA antibody. Each immunoprecipitate was subjected to SDS‐PAGE, followed by WB using anti‐GFP and anti‐HA antibodies. (**g**) Cell lysates from HEK293 cells expressing GFP-JRAB-CH + LIM WT or mutant (L197E/V198E/R200E) together with HA‐filaminA were immunoprecipitated (IP) with anti‐HA antibody. Each immunoprecipitate was detected as described in (**f**). (**d**–**g**) Full-length blots are presented in Supplementary Fig. S6.

We previously reported structural models of JRAB-C in complex with Rab13 and with JRAB-N (LIM)^8^. At around the same time, another group reported the X-ray crystal structure of the MICAL C-terminal–like protein (MICAL-CL) and Rab8A^15^. Sequence alignments showed high similarities between MICAL-CL and JRAB-C and between Rab8A and Rab13 (see Materials and Methods), and Rab8A also interacts with JRAB/MICAL-L2^5^; therefore, we tried to construct the structural model of JRAB-C by homology modeling based on the structure of MICAL-CL, and the accuracy of the present model was higher than that of previous one (Fig. 3a, right).

To develop a reliable complex structural model between JRAB-N and JRAB-C, we performed a docking simulation of the structure model of the regions containing the LIM domain (aa 181–250) and that of the C-terminal region (aa 827–984), which was constructed by the homology modeling as described above, using the docking server, ClusPro 2.0^16^. According to the result obtained from HDX-MS, we specified the residues from G189 to L207 of ZF1 as being “attraction” in the docking, which indicates that interactions involving these ZF1 residues were positively evaluated in the scoring step. Among the residues that appeared frequently in the binding sites of the complex models obtained from the docking simulation, we selected the residues M884, W887, Q895, and L898 in helix 2 of JRAB-C as candidates for the residues involved in the association with ZF1 (Fig. 3a, left). In the same way, the frequently appeared LIM residues in the binding sites of the complex models, L197, V198, and R200, were also detected (Fig. 3b, left).

To verify the findings of the docking simulation, we prepared three kinds of mutants: in two of the mutants, two residues in JRAB-C were replaced with lysine, a positively charged amino acid (M884K and W887K or Q895K and L898K) (Fig. 3c, left) and in the third mutant, three residues in JRAB-ZF1 were replaced with glutamic acid, a negatively charged amino acid (L197E, V198E, and R200E) (Fig. 3c, right). It should be noted that the surface properties of JRAB-C model are electrostatically negative and hydrophobic (Fig. 3a, right) and those of JRAB-LIM model are electrostatically positive and hydrophobic (Fig. 3b, right). With these mutants, we performed pull-down assays using GST-JRAB-CH + LIM and GST-JRAB-C. Neither GFP-JRAB-C (M884K/W887K) nor GFP-JRAB-C (Q895K/L898K) was pulled down by GST-JRAB-CH + LIM (Fig. 3d). Similarly, GFP-JRAB-CH + LIM (L197E/V198E/R200E) was not pulled down by GST-JRAB-C (Fig. 3e). To confirm these mutants are still capable of binding something outside of the mutated region, we performed immunoprecipitation assays. Both JRAB-C mutants still have ability to interact with the dominant active form of Rab13 (Rab13DA) (Fig. 3f). The triple mutations in JRAB-ZF1 have no effect on the interaction with filaminA (Fig. 3g). Thus, these biochemical results indicate that M884, W887, Q895, and L898 in helix 2 of JRAB-C and L197, V198, and R200 in JRAB-ZF1 are specifically required for the interaction between JRAB-LIM and JRAB-C.

### Interaction between ZF1 domain and C-terminal domain of JRAB/MICAL-L2 regulates the association of JRAB-LIM with F-actin

**Figure 4:**
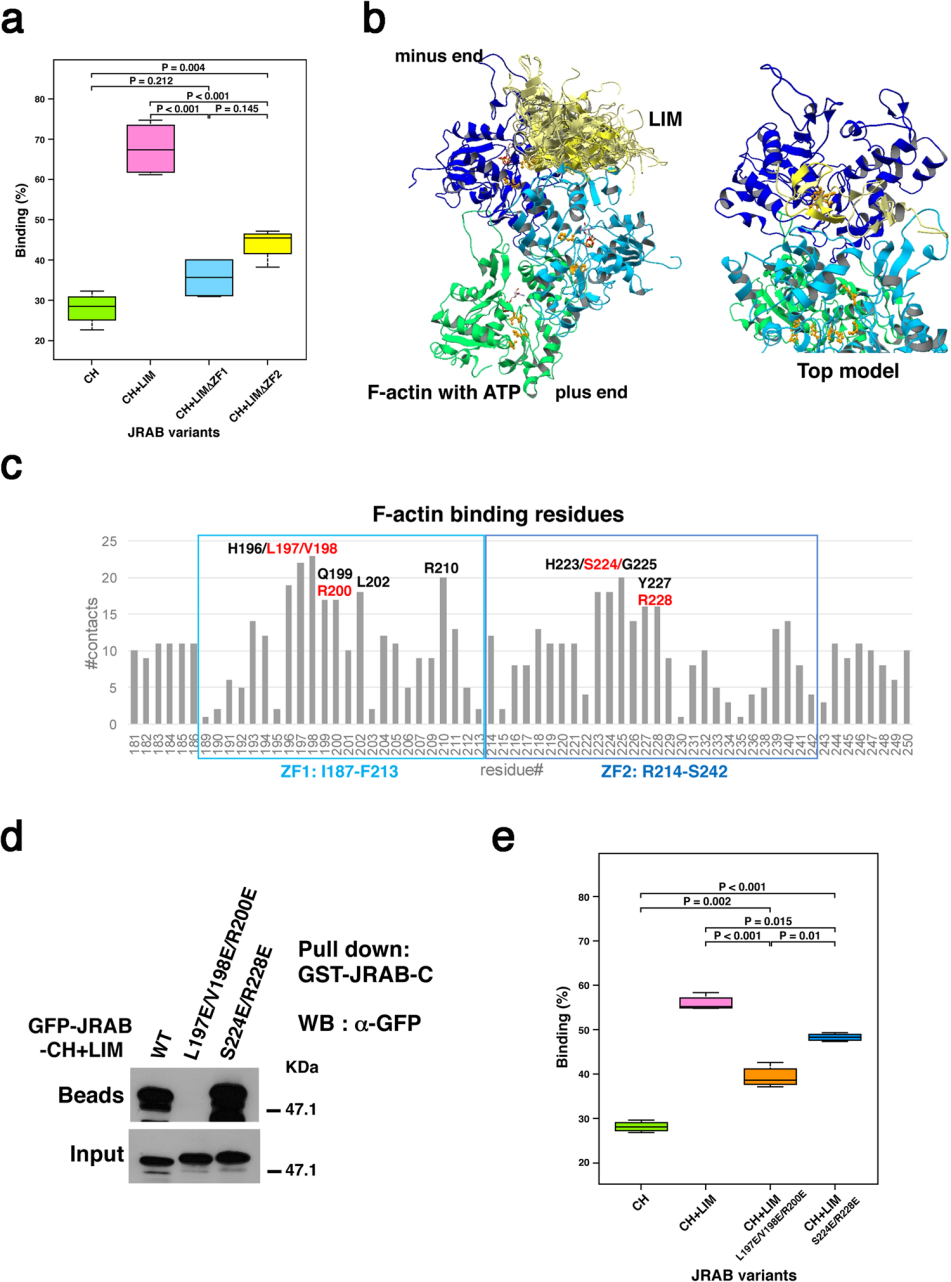
Three residues of the ZF1 domain are responsible for not only the intramolecular interaction, but also the association of JRAB-LIM with F-actin. (**a**) F-actin binding assay using the indicated JRAB variants. Graph shows the ratio of each recombinant protein in the pellet vs. total recombinant protein in the pellet and supernatant. Differences among groups were tested by ANOVA with Tukey’s post-hoc multiple comparison test. Differences were considered significant when *p* < 0.05. See also Supplementary Fig. S2a. (**b**) Docking simulation between JRAB-LIM and F-actin. The merge of 24 docking models in which LIM binds both the first (blue) and second (cyan) actin monomers from the minus end are shown, along with the top model among them. H73, P109, and H161, which are involved in P_i_-release, are colored orange. (**c**) Numbers of contacts of the residues in JRAB-LIM ZF1 and ZF2 regions with F-actin; the residues that appeared frequently are indicated. The residues colored in red were selected as candidates for the mutation sites to break the interaction with F-actin. To alter the positively charged surface in the putative actin-binding site on LIM to a negatively charged surface, L197, V198, and R200 in the ZF1 region, and S224 and R228 in the ZF2 region were replaced with Glu (E) residues. (**d**) HEK293 cell lysates containing GFP-JRAB-CH + LIM WT or mutant (L197E/V198E/R200E or S224E/R228E) were subjected to pull-down assays using GST-JRAB-C. The pulled-down protein was detected by WB using anti-GFP antibody. Full-length blot is presented in Supplementary Fig. S7. (**e**) F-actin binding assay using His-JRAB-CH + LIM mutants (L197E/V198E/R200E and S224E/R228E). Graph shows values as described in (**a**). Differences among groups were tested by ANOVA with Tukey’s post-hoc multiple comparison test. Differences were considered significant when *p* < 0.05. See also Supplementary Fig. S2c.

We previously showed that JRAB-LIM binds to F-actin^7^. In this study, we sought to determine how the LIM domain associates with F-actin and regulates actin cytoskeletal reorganization. First, we investigated whether ZF1 or ZF2 associates with F-actin. For this purpose, we prepared His-tagged recombinant JRAB-CH + LIM WT, -CH + LIMΔZF1, -CH + LIMΔZF2 and -CH alone, and performed *in vitro* F-actin binding assays. Most of the His-JRAB-CH + LIM WT interacted with F-actin in the pellet fraction as previously reported^7^, whereas the amounts of His-JRAB-CH + LIMΔZF1 and His-JRAB-CH + LIMΔZF2 precipitated with F-actin were less than that of His-JRAB-CH + LIM WT (Fig. 4a and Supplementary Fig. S2a). His-JRAB-CH was used as a negative control in these experiments. This result indicates that both ZF1 and ZF2 contribute to the interaction of JRAB-LIM with F-actin.

Next, in order to predict LIM residues that bind to F-actin, we performed a docking simulation of the ATP-bound F-actin (trimer) and LIM models using ClusPro 2.0^16^. Among the F-actin–LIM complex models, we focused on 24 models in which LIM binds to both the first and second actin monomers from the minus end of the F-actin trimer (Fig. 4b). This is because we previously showed that the LIM domain is involved in the inhibition of F-actin depolymerization by biochemical analyses^7^. We analyzed F-actin–LIM interactions in the complex models and found seven LIM residues in the ZF1 domain and five residues in the ZF2 domain that appeared very frequently in the binding sites (Fig. 4c). Among them, L197, V198, and R200 are the residues responsible for the interaction with JRAB-C, as described above (see Fig. 3e). To confirm the involvement of three residues of ZF1 in the interaction between F-actin and ZF1, we used the mutant JRAB-CH + LIM (L197E/V198E/R200E) (Fig. 3c, right). As to ZF2, we selected S224 and R228 as candidates for the mutation sites to break the interaction with F-actin and prepared another mutant, in which two residues were replaced with glutamic acid, a negatively charged amino acid (S224E/R228E) (Supplementary Fig. S2b). It should be noted that the double mutations (S224E and R228E) have no effect on the interaction between JRAB-CH + LIM and JRAB-C, although JRAB-CH + LIM (L197E/V198E/R200E) did not interact with JRAB-C (Fig. 4d). Using the *in vitro* F-actin binding assay, we examined the interaction between F-actin and the mutated recombinant proteins, JRAB-CH + LIM (L197E/V198E/R200E) and JRAB-CH + LIM (S224E/R228E). Both proteins interacted with F-actin, but to a lesser extent than JRAB-CH + LIM WT (Fig. 4e and Supplementary Fig. S2c). These results suggest that the residues L197, V198, and R200 of ZF1 and the residues S224 and R228 of ZF2 are necessary for the association of JRAB-LIM with F-actin, and that the three residues of ZF1 are also necessary for the interaction between JRAB-LIM and JRAB-C. Together, JRAB-LIM binds to F-actin via both ZF1 and ZF2, and JRAB-ZF1 fine-tunes actin cytoskeletal rearrangement via the intramolecular interaction with JRAB-C.

### JRAB ZF1 free from the JRAB C-terminus is required for the cellular function of JRAB/MICAL-L2 in the open form

**Figure 5:**
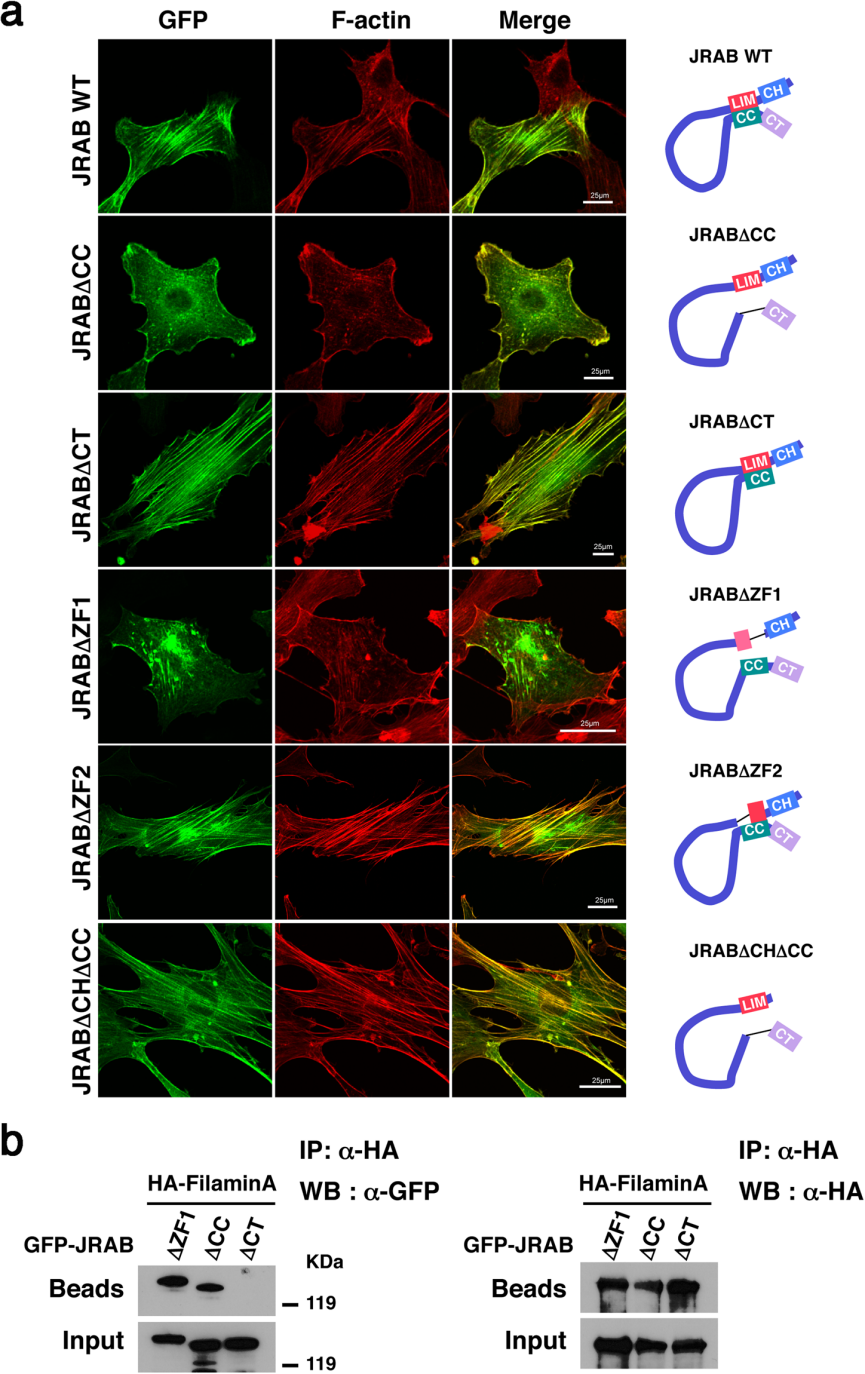
Not only the interaction between JRAB-CH and filamin, but also JRAB ZF1 free from JRAB-C, is required for the function of open-form JRAB/MICAL-L2. (**a**) NIH3T3 cells expressing GFP‐JRAB WT or the indicated GFP‐JRAB variant were fixed and processed for rhodamine–phalloidin staining (red). Bar, 25 μm. Results shown are representative of at least three independent experiments. Schematic indicates the conformation of JRAB variants (right). (**b**) Cell lysates from HEK293 cells expressing GFP‐JRABΔZF1, ‐JRABΔCC, or ‐JRABΔCT together with HA‐filaminA were immunoprecipitated (IP) with anti‐HA antibody. Each immunoprecipitate was subjected to SDS‐PAGE, followed by WB with anti‐GFP and anti‐HA antibodies. Full-length blots with multiple exposures are presented in Supplementary Fig. S7.

Next, to examine the role of ZF1 domain in the cellular function, we prepared an NIH3T3 mouse embryo fibroblast cell line expressing GFP-JRABΔZF1 using a retroviral expression system. In a previous study, we used an mCherry-tagged construct to show that JRABΔCC and JRABΔCT influence individual cellular morphology and actin structure^10^. Consistent with our previous data, the formation of stress fibers was increased by expression of GFP-JRABΔCT compared to GFP-JRAB WT (Fig. 5a) or GFP as negative control (Supplementary Fig. S3). On the other hand, GFP-JRABΔCC induced membrane ruffles. GFP-JRABΔCT and GFP-JRABΔCC localized along stress fibers and at the edges of ruffles, respectively. GFP-JRABΔZF1, which is presumed to adopt the open form, induced very faint and disarrayed stress fibers relative to cells expressing GFP-JRABΔCT (Fig. 5a). Moreover, membrane ruffles were rarely observed in the cells expressing GFP-JRABΔZF1 compared to the cells expressing GFP-JRABΔCC (Fig. 5a), and even though it was barely detectable, the recruitment of GFP-JRABΔZF1 to the edge of the ruffles was disturbed. These findings indicate that GFP-JRABΔZF1 adopts the open form, but does not have the effects on cellular morphology and actin cytoskeletal reorganization that were observed in cells expressing GFP-JRABΔCC. By contrast, the stress fibers were observed in the cells expressing GFP-JRABΔZF2 (aa 1–213 + 242–1009) (Supplementary Fig. S1) as well as the cells expressing GFP-JRAB WT (Fig. 5a). It should be noted that GFP-JRABΔZF2 is presumed to adopt the closed form via the interaction between ZF1domain and CC domain.

Previously, we identified filamin as a specific binding partner for the open form of JRAB/MICAL-L2^10^. JRAB/MICAL-L2 binds to filamin via its CH domain, and this interaction is necessary for cell spreading accompanied by formation of peripheral ruffles. As we showed in that study and Fig. 5a, GFP-JRABΔCHΔCC (aa 139–805 + 913–1009) (Supplementary Fig. S1), a mutant lacking the filamin-binding domain, did not induce formation of peripheral ruffles like GFP-JRABΔCC, even though it is maintained in the open form. Hence, we examined the interaction between JRABΔZF1 and filamin. Immunoprecipitation assays revealed that GFP-JRABΔZF1 and GFP-JRABΔCC were coimmunoprecipitated with HA-filaminA, but GFP-JRABΔCT was not (Fig. 5b). These results show that not only the interaction between JRAB/MICAL-L2 and filamin, but also the LIM domain itself, especially the ZF1 domain, is necessary to fulfill the roles of open-form JRAB/MICAL-L2.

## Discussion

Previously, we reported that JRAB/MICAL-L2 changes its conformation from closed to open, dependent upon association with Rab13^7,8,10,12^. Moreover, we provided structural models for JRAB-C-Rab13 and JRAB-C-JRAB-LIM using a combination of biochemical analysis and bioinformatics^8^. Around the same time, Müller’s group reported the X-ray crystal structure of the complex of MICAL C-terminal–like protein (MICAL-CL) and Rab8A^15^. According to alignments of MICAL-CL and the C-terminus of JRAB/MICAL-L2, and Rab8A and Rab13, the amino acid residues of MICAL-CL involved in binding to Rab8A are conserved in the C-terminus of JRAB/MICAL-L2, and the residues of Rab8A involved in binding MICAL-CL are also conserved in Rab13. Homology modeling of the JRAB–Rab13 complex revealed that a putative Rab13 binding site is located opposite to the LIM binding site (Supplementary Fig. S4a). This raises the question of how Rab13 kicks out the LIM domain, resulting in the conformational change of JRAB/MICAL-L2. The inconsistency could be explained by the finding that some MICAL family members have two similar Rab-binding sites, resulting in association with two Rab proteins simultaneously at separate sites, e.g. MICAL-1 can bind two molecules of Rab10 with different binding affinities^15^, where one (high-affinity site) is the similar region to the predicted Rab13 binding site and another (low-affinity site) overlaps with the region corresponding to the predicted LIM binding site on JRAB-C (Supplementary Fig. S5). The complex model between JRAB-C and two Rab13 could explain not only the competition mechanism between LIM and Rab13, but also the consistency between the structural model and biochemical data shown in our previous study, where Rab13 binds to the region from helix 2 to helix 5 plus loop 4 in JRAB-C^8^. In addition, there are high sequence similarities between Rab10 and Rab13, as well as between MICAL-1 and JRAB/MICAL-L2. We therefore suppose that the complex model of JRAB-C with two molecules of Rab13 is one of the possible complex models between JRAB-C and Rab13 (Supplementary Fig. S4b), although further verification may be required. It should be noted that the model does not contradict the finding that JRABΔCT lacking the CT domain (aa 913–1009) adopts the closed form. The reasons are as follows. First, the binding sites of two molecules of Rab13 on JRAB/MICAL-L2 corresponding to the binding sites of two molecules of Rab10 on MICAL-1 are included in the CT domain of JRAB/MICAL-L2 (Supplementary Fig. S5). Second, JRAB-C constructs a three-helix bundle, which requires a tight bundling of the long three helices to maintain the conformation including the Rab13 binding sites as shown in Fig. 3a. Therefore, JRAB/MICAL-L2 without CT domain may not construct the three-helix bundle structure. From these, JRABΔCT would not bind to two molecules of Rab13, resulting in its closed conformation.

F-actin associates with the LIM domain and the latter part of disordered region of JRAB/MICAL-L2^7^. In this study, we analyzed F-actin–JRAB-LIM interactions by performing docking simulations of F-actin (trimer) and JRAB-LIM models. Among the obtained F-actin–LIM complex models, we selected 24 models in which LIM binds to both the first and second actin monomers from the minus end of the F-actin trimers. We made this selection for several reasons. First, we previously demonstrated that the LIM domain is involved in inhibition of actin depolymerization^7^. Second, actin monomers tend to be depolymerized at the minus end of F-actin, in part because ATP tends to form ADP + P_i_ at the minus end, and actin-ADP has a higher affinity than actin-ATP for the actin-severing proteins ADF and cofilin^17–19^. Third, P_i_ release is involved in the depolymerization of the first actin^19–21^, and the P_i_-release cavity in the first actin is located close to the second actin. The docking model indicates that ZF1 and ZF2 within the LIM domain bind to first and second actin molecules, respectively. This is supported by the results of the *in vitro* F-actin binding assay. Together, these findings show that the LIM domain promotes actin cytoskeletal reorganization (inhibition of depolymerization of F-actin) via both ZF1 and ZF2, whereas only ZF1 contributes to the mode of action of the whole LIM domain through the association with JRAB-C. Additionally, it is worth noting that the same residues of ZF1 are involved in the associations with F-actin and JRAB-C.

MICAL family is consisting of MICAL proteins and MICAL-like proteins^22^. MICAL proteins, MICAL-1, -2, and -3 contain FAD domains, which have a redox potential, whereas MICAL-like proteins including JRAB/MICAL-L2 lack this domain. The FAD domain of the MICAL proteins has been extensively studied, revealing the regulatory mechanism of actin cytoskeletal rearrangements^23,24^. According to a widely held view, MICAL proteins oxidize methionine residues of actin molecules, leading to F-actin depolymerization^25^. Here, we described the mechanism by which the LIM domain of a MICAL-like protein, JRAB/MICAL-L2, participates in inhibition of F-actin depolymerization.

In our previous study, we showed that NIH3T3 cells expressing open-form JRAB/MICAL-L2 exhibits peripheral ruffle without stress fibers, whereas expression of closed-form JRAB/MICAL-L2 promotes the formation of stress fibers^10^. We also showed that the actin-binding protein filamin preferentially binds to open-form JRAB/MICAL-L2, and the JRAB/MICAL-L2–filamin complex induces peripheral ruffles, leading to cell spreading^10^. In this work, to examine the role of the LIM domain itself in actin cytoskeletal reorganization via open-form JRAB/MICAL-L2, we generated a new JRAB/MICAL-L2 mutant lacking ZF1. The mutant is maintained in the open form and has the ability to interact with filamin. In NIH3T3 cells expressing JRABΔZF1, faint and disarrayed stress fibers were observed and peripheral ruffles were much less abundant than in cells expressing JRABΔCC. Even though peripheral ruffles were observed, the recruitment of JRABΔZF1 to the ruffles was impaired. Cell biological analysis revealed that the LIM domain itself as well as the interaction between JRAB/MICAL-L2 and filamin is essential for the full function of open-form JRAB/MICAL-L2. Overall, our results shed new light on how LIM domain connects the conformational dynamics of JRAB/MICAL-L2 to multiple functions.

## Materials and Methods

### Ethical statement

All experiments were conducted according to protocols reviewed and approved by the Committee for Safe Handling of Living Modified Organisms (Permission number 30–96) in Tokushima University.

### Plasmid construction

For the ΔLIM mutant, the ΔZF1 mutant and the ΔZF2 mutant of mouse JRAB/MICAL-L2, the coding regions for amino acids (aa) 1–138 and 261–1009, aa 1–185 and 213–1009, and aa 1–213 and 242–1009, were PCR-amplified using pCIneo-HA-JRAB/MICAL-L2 as a template. The products were ligated and subcloned into vector pEGFP-C1. pEGFP-JRAB WT, -JRABΔCC, and -JRABΔCT were generated as reported previously^8^. pcDNAHisMax-JRAB WT, -JRABΔCC, and -JRABΔCT were generated as described previously^8^. JRAB/MICAL-L2 truncated mutants; CH + LIMΔZF1 (aa 1–185 + 213–260) and CH + LIMΔZF2 (aa 1–213 + 242–260) were amplified by PCR using pEGFP-JRABΔZF1 and pEGFP-JRABΔZF2 as a template, respectively. The products were subcloned into pEGFP-C1 or pRSET-A. pEGFP-JRAB-CH + LIM, -JRAB-CH, -JRAB-LIM, -JRAB-C, pRSET-A-JRAB-CH + LIM, -JRAB-CH, pGEX-6P-1-JRAB-CH + LIM and -JRAB-C were described previously^7^. pEGFP-JRAB-C mutants (M884K/W887K and Q895K/L898K) and -JRAB-CH + LIM mutants (L197E/V198E/R200E and S224E/R228E) were constructed using a QuikChange Lightning Multi Site-Directed Mutagenesis Kit (Agilent Technologies Inc). cDNAs encoding JRAB-CH + LIM mutants (L197E/V198E/R200E and S224E/R228E) were subcloned into pRSET-A. pCIneo-HA-Rab13DA (Q67L) and –filaminA were described previously^1,10^. To produce retrovirus-expressing GFP-tagged proteins, we generated pMX-EGFP-JRAB WT, -JRABΔCC, -JRABΔCT, -JRABΔZF1, -JRABΔZF2, and -JRABΔCHΔCC as previously described^7,10,26^. All plasmids constructed in this study were sequenced on an ABI Prism 3100 genetic analyzer (Applied Biosystems). Schematic presentation of the molecular structures of JRAB/MICAL-L2 and all constructs was shown in Figs 1a and 2a, and Supplementary Fig. S1.

### Recombinant retrovirus preparation/infection and cell staining

Vector pMX-EGFP containing cDNA of JRAB/MICAL-L2 or its mutant derivatives was transfected into PLAT-E cells as described previously^27^. After a 48-h transfection, culture supernatant was collected and passed through a 0.45-μm filter prior to infection of NIH3T3 cells. The cells expressing GFP-fused protein(s) were stained by rhodamine–phalloidin as described previously^10^.

### Pull-down assays

Pull-down assay was performed as described previously^7,8,10^. HEK293 cells were seeded in 60-mm dishes and transfected the following day with appropriate amount of each plasmid using PEI-MAX transfection reagent^28^. After a 48-h incubation, the cells were lysed and then centrifuged to remove the debris. Each supernatant was incubated with purified GST-JRAB-C or -CH + LIM attached to glutathione–Sepharose beads. The beads were then washed and resuspended in sodium dodecyl sulfate (SDS) sample buffer. Comparable amounts of the proteins that remained associated with the beads were separated by SDS–polyacrylamide gel electrophoresis (PAGE). The fraction of target protein bound to the beads was determined by western blotting using an anti-GFP antibody (Invitrogen) as described^7,10^.

### Immunoprecipitation

Immunoprecipitation was performed as described previously^7,10^. Forty-eight hours after transfection, HEK293 cells were lysed and then centrifuged to remove the debris. An aliquot of the supernatant was used to verify expression of the indicated proteins. The rest of the supernatant was mixed with Protein G–Sepharose FF beads (GE Healthcare Biosciences) linked to anti-HA monoclonal antibody (12CA5; Roche Diagnostics). The beads were washed and then resuspended in SDS sample buffer. The immunoprecipitates were subjected to western blotting using anti-HA (3F10; Roche Diagnostics) and anti-GFP antibodies as described previously^7^.

### *In vitro* F-actin binding assay

*In vitro* F-actin binding assay was performed as described previously^7^. The His-tagged recombinant proteins were expressed in *E*. *coli* and purified using TALON metal affinity resin (Clontech) as described previously^7^. F-actin (Cytoskeleton) (23 μM stock) was incubated with purified recombinant proteins. The mixture was ultracentrifuged, and the supernatant and pellet were subjected to SDS-PAGE followed by Coomassie Brilliant Blue (CBB) staining. The quantitative analyses were performed by the Image Lab software in the Gel Doc EZ system (Bio-Rad). Differences among groups were tested by ANOVA followed by Tukey’s post-hoc multiple comparison test. Differences were considered significant when *p* < 0.05.

### Purification of recombinant proteins from HEK293 cells

HEK293 cells were seeded on 100-mm dishes and transfected the following day with 12 μg of each plasmid using PEI-MAX transfection reagent^28^. After a 48-h incubation, the cells were lysed and then centrifuged to remove the debris. Supernatants collected from 30 dishes were applied to cOmplete resin column (Roche). The proteins remained in the column were eluted and subjected to SDS-PAGE. The samples were concentrated to 1.5 mg/ml, and then 500 μl of protein solution was applied to a Superdex 200 PC 3.2/30 (GE Healthcare) column (2.6 × 6.6 cm). The fractions were collected and subjected to SDS-PAGE, followed by CBB staining.

### Hydrogen/deuterium exchange experiment

HDX-MS analysis was performed on an HDx-3 PAL system (LEAP Technologies) with Ultimate 3000 RSLC nano (Thermo Fisher Scientific). Protein was 10-fold diluted and labeled with H_2_O or D_2_O buffer by chiller syringe at 10 °C for the indicated exchange times, and then quenched with an equal volume of 2 M guanidine HCl 100 mM citric acid (pH 2.3) at 0 °C for 30 sec. The quenched sample was injected into the sample loop and transferred to a pepsin column (Poroszyme Immobilized Pepsin Cartridge, 2.1 × 30 mm, Thermo Fisher Scientific) with 0.1% FA at flow rate of 50 μl/min by loading pump. After digestion, peptides were trapped on the trap column (Acclaim PepMap300 C18 5 μm, 1 × 15 mm, Thermo Fisher Scientific) and separated on an analytical column (Hypersil Gold, 1 × 50 mm, 1.9 μm, Thermo Fisher Scientific) with a 10–25% gradient of 90% ACN / 0.1% FA for 10 min at a flow rate of 45 μl/min. Eluted peptides were detected on a Q Exactive mass spectrometer (Thermo Fisher Scientific) with resolution of 70,000 for MS scan and 17,500 for MS/MS scan. Peptides were identified based on MS/MS spectra using Proteome Discoverer 2.2 (Thermo Fisher Scientific) and processed to validate H/D exchange rate using HDExaminer 2.3 (Sierra Analytics).

### Modeling of JRAB-C and JRAB-LIM domains

Structural models of JRAB-C and JRAB-LIM domains were predicted based on the crystal structures of their homologous proteins. As a template, we used the crystal structure of human MICAL-CL, the homolog of JRAB-C, in complex with Rab8A (Protein Data Bank (PDB) ID: 5SZI) due to the similarity between the JRAB-C and MICAL-CL sequences (80.8%). First, the sequence of JRAB-C (the C-terminal domain of JRAB/MICAL-L2, UniProt ID of JRAB: Q3TN34) was aligned to that of human MICAL-CL (UniProt ID: Q6ZW33) using Ssearch with the MIQS matrix^29^, with the gap-open penalty set to −13. Then, the structural model was constructed based on the alignment and the crystal structure of human MICAL-CL (the subunit structure with chain B in 5SZI) using Modeller 9.19^30^. Similarly, the sequence of the LIM domain in JRAB/MICAL-L2 was aligned to that of human MICAL-1 (UniProt ID: Q8TDZ2), which is 86.7% similar at the sequence level^29^. The structure was modeled based on the alignment and structure of the LIM domain of MICAL-1 (PDBID: 2CO8)^30^ by Modeller.

### Structural modeling of Rab13 and the JRAB-C–Rab13 complex

The Rab13 structure was modeled as described above for JRAB-C. Here, the structure of human Rab8A (the subunit structure with chain A in 5SZI) was used as the template due to the sequence similarity between mouse Rab13 and human Rab8A^29^ (94.1%; UniProt IDs: Q9DD03 and P61006, respectively). To obtain a complex model between Rab13 and JRAB-C, the models of Rab13 and JRAB-C were superimposed onto the structures of Rab8A and MICAL-CL (PDBID: 5SZI), respectively, using CCP4i^31^.

### Modeling of the complex between JRAB-C and two Rab13 molecules

The structural models of JRAB-C and Rab13, constructed as described above, were superimposed onto the structures of MICAL-1 and Rab10 (PDBID: 5LPN), respectively, using CCP4i^31^. It should be noted that the Rab13 structure model was superimposed onto both of the Rab10 molecules in the complex structure.

### JRAB-C–JRAB-LIM docking simulation

To obtain information about interactions between JRAB-C and JRAB-LIM domains, we performed a docking simulation of JRAB-C and JRAB-LIM models using ClusPro 2.0^16^. We specified the residues from G189 to L207 in the ZF1 domain as being “attraction” in the docking, which indicates that interactions involving these LIM residues were positively evaluated in the scoring step. Among the residues that appeared frequently in the binding sites of the complex models obtained from the docking, we selected the residues M884, W887, Q895, and L898 in helix 2 of JRAB-C as candidates for mutation sites to inhibit the JRAB-C–JRAB-LIM interaction. It should be noted that because the surface properties of the JRAB-C model were electrostatically negative and hydrophobic, we selected negatively charged, hydrophobic, or polar residues to break the interaction. The LIM residues frequently appearing in the binding sites of the complex models (L197, V198, and R200) were also detected. These positively charged and hydrophobic residues are likely to interact with the selected JRAB-C residues described above.

### Surface properties of JRAB-C and JRAB-LIM mutants

The electrostatic properties and hydrophobicity of the molecular surfaces of the structural models were calculated as follows. Electrostatic potential was calculated for each atom using the program SCB^32^. The hydrophobic property was calculated according to the hydropathy value peculiar to the side-chain of a residue^33^. The electrostatic potential and hydrophobicity values were then assigned to the molecular surface of the protein, calculated using the program MSP^34^. We also calculated these surface properties for the mutant structures in order to examine changes in surface properties caused by introduction of the mutations. All figures were drawn using the interactive molecular viewer jV^35^.

### Construction of a model of ATP-binding F-actin

The F-actin structure, as determined by electron microscopy (EM) (PDBID: 3J8I), contains five actin monomers and ADP molecules with Mg ions. To construct a model of ATP-binding F-actin, we superimposed the crystal structure of actin monomer in complex with ATP and Mg (PDBID: 1YAG) onto each of the five actin monomers in the F-actin EM structure using CCP4i^31^; the actin residues from E4 to F31, I71 to D179, and N225 to F375 were superimposed to obtain a good model. We then replaced the original ADP and Mg molecules with the ATP and Mg molecules in the crystal structure.

The F-actin EM structure with ATP and Mg was energetically minimized and equilibrated using the MD simulation program Gromacs^36^. The snapshot after energy minimization for approximately 50,000 steps and equilibration for 1 ns (500 ps NVT, followed by 500-ps NPT steps), was used as the model of ATP-binding F-actin in the subsequent docking simulation between F-actin and LIM. To decrease the computational cost of the MD and subsequent docking simulations, we performed the energy minimization and equilibration for the F-actin structure containing only the first three of the five actin monomers.

### F-actin–JRAB-LIM docking simulation

The docking simulation of the ATP-binding F-actin (trimer) and JRAB-LIM models was performed using ClusPro 2.0^16^. Among the F-actin–LIM complex models we obtained, we selected 24 models in which LIM binds to both the first and second actin monomers from the minus end of the F-actin trimer. We analyzed actin–JRAB-LIM interactions in the 24 complex models and found seven residues of JRAB-ZF1 and five residues of JRAB-ZF2 that appeared in the binding sites very frequently. Among them, we selected L197, V198, R200, S224, and R228 and prepared a triple mutant (L197E/V198E/R200E) and double mutant (S224E/R228E) to examine their effects on the association with F-actin. The structures of the mutants were predicted using Scwrl 4.0^37^ based on the LIM model structure.

## Supplementary information

Supplementary information

## Data Availability

All data generated or analyzed during this study are included in this published article.
