# Supplementary material for: Actin Cytoskeletal Reorganization Function of JRAB/MICAL-L2 Is Fine-tuned by Intramolecular Interaction between First LIM Zinc Finger and C-terminal Coiled-coil Domains
Source: Sci Rep. 2019 Sep 5;9:12794. doi: 10.1038/s41598-019-49232-8 (PMC6728388; doi:10.1038/s41598-019-49232-8)
Supplement: Supplementary file 1 — Supplementary information [file 41598_2019_49232_MOESM1_ESM.pdf]

Supplementary Information

Actin Cytoskeletal Reorganization Function of JRAB/MICAL-L2 Is Fine-tuned by Intramolecular Interaction between First LIM Zinc Finger and C-terminal Coiled-coil Domains

Kazuhisa Miyake[1], Ayuko Sakane[1,2], Yuko Tsuchiya[3,4], Ikuko Sagawa[5], Yoko Tomida[1,6], Jiro Kasahara[6], Issei Imoto[7,8], Shio Watanabe[9], Daisuke Higo[9], Kenji Mizuguchi[4], and Takuya Sasaki[1]

[1]Department of Biochemistry, Tokushima University Graduate School of Medical Sciences, Tokushima 770-8503, Japan; [2]Department of Interdisciplinary Researches for Medicine and Photonics, Institute of Post-LED Photonics, Tokushima University, Tokushima 770-8506, Japan; [3]Intelligent Bioinformatics Research Team, Artificial Intelligence Research Center, The National Institute of Advanced Industrial Science and Technology, Tokyo 135-0064, Japan; [4]National Institutes of Biomedical Innovation, Health and Nutrition, Ibaraki 567-0085, Japan; [5]Support Center for Advanced Medical

Sciences, Tokushima University Graduate School of Biomedical Sciences, Tokushima 770-8503, Japan; [6]Department of Neurobiology and Therapeutics, Faculty of Pharmaceutical Sciences, Tokushima University, Tokushima 770-8503, Japan; [7]Division of Molecular Genetics, Aichi Cancer Center Research Institute, Nagoya 464-8681, Japan; [8]Department of Cancer Genetics, Nagoya University Graduate School of Medicine, Nagoya 466-8550, Japan; [9]Thermo Fisher Scientific, Chromatography & MS Department, Application Group, LC-MS, Yokohama 221-0022, Japan.

Supplementary Figure legends

Supplementary Figure S1. Structures of the full-length JRAB/MICAL-L2 and JRAB/MICAL-L2 variants. CH: calponin homology domain, LIM: LIM domain, ZF1: first zinc finger domain, ZF2: second zinc finger domain, CC: coiled-coil domain, CT: the C-terminal region, which follows the CC domain. Numbers represent amino acid positions.

Supplementary Figure S2. JRAB-LIM binds to F-actin via two zinc finger domains. (a)(c) Each recombinant protein was incubated with F-actin and centrifuged at 125,000 × *g*. Aliquots of pellet (P) and supernatant (S) were subjected to SDS-PAGE, followed by CBB staining. Asterisk, actin; arrowhead, indicated recombinant protein. Each image is representative of four independent experiments. Statistical analyses of the results are shown in Fig. 4a (a) and 4e (c). Full-length gels are presented in Supplementary Fig. S7. (b) Structure model of JRAB-LIM double mutant (S224E/R228E), in which the side-chain conformations of the mutated residues were predicted using Scwrl 4.0.

Supplementary Figure S3. NIH3T3 cells expressing GFP as a negative control for the cells expressing GFP-JRAB variants in Figure 5a. Cells expressing GFP were fixed and processed for rhodamine–phalloidin staining (red). Bar, 25 μm.

Supplementary Figure S4. Structural models of the complex between JRAB-C and Rab13. (a) Model of the JRAB-C–Rab13 complex based on the MICAL-CL–Rab8A complex (PDBID: 5SZI) (center). Putative helix regions (helix 1 [h1] – helix 5 [h5]) in JRAB-C are shown in different colors, and Rab13 is shown in pink. LIM is shown in yellow. The left and right figures show the surface properties of JRAB-C and Rab13; the Rab13 and JRAB-C binding sites on JRAB-C and Rab13 are highlighted. Red and blue surfaces indicate electrostatically negative and positive regions, respectively. Hydrophobicity is indicated in yellow. Surface properties were calculated as described in the Method section. (b) Model of the complex of JRAB-C and two Rab13 molecules, based on the complex of MICAL-1 with two Rab10 molecules (PDBID: 5LPN), in a different orientation. In this model, one of the two Rab13 molecules (Rab13-1) mainly binds to the helix 3 (h3), helix 4 (h4), and helix 5 (h5) regions of JRAB-C, whereas the

other (Rab13-2) mainly binds to helix 2 (h2), helix 4 (h4), and helix 5 (h5), which are on the opposite side of the Rab13-1 binding site.

Supplementary Figure S5. Sequence alignment between the C-terminal region of MICAL-1 and that of JRAB/MICAL-L2. The sequence of the C-terminal domain of JRAB (Uniprot ID: Q3TN34) was aligned into that of human MICAL-1 (Uniprot ID: Q8TDZ2) by using Ssearch with MIQS matrix, of which sequence similarity is 82.3%. Red and yellow colored residues indicate the Rab10-2 binding site (low affinity) and the Rab10-1 binding site (high affinity), respectively. In the sequence of JRAB-C, the residues belonging to the five helix regions are colored blue (helix 1), light blue (helix 2) with an underline (helix 3), and sky blue (helix 4) with an underline (helix 5).

Supplementary Figure S6. Full-length images of blots. Uncropped images of scanned blots in Figure 2a, 2b, 3d, 3e, 3f, and 3g. The Square indicates each cropped area.

Supplementary Figure S7. Full-length images of blots and gels. Uncropped images of scanned blots or gels in Figure 4d, 5b, and Supplementary Figure S2a and S2c. The Square indicates each cropped area.

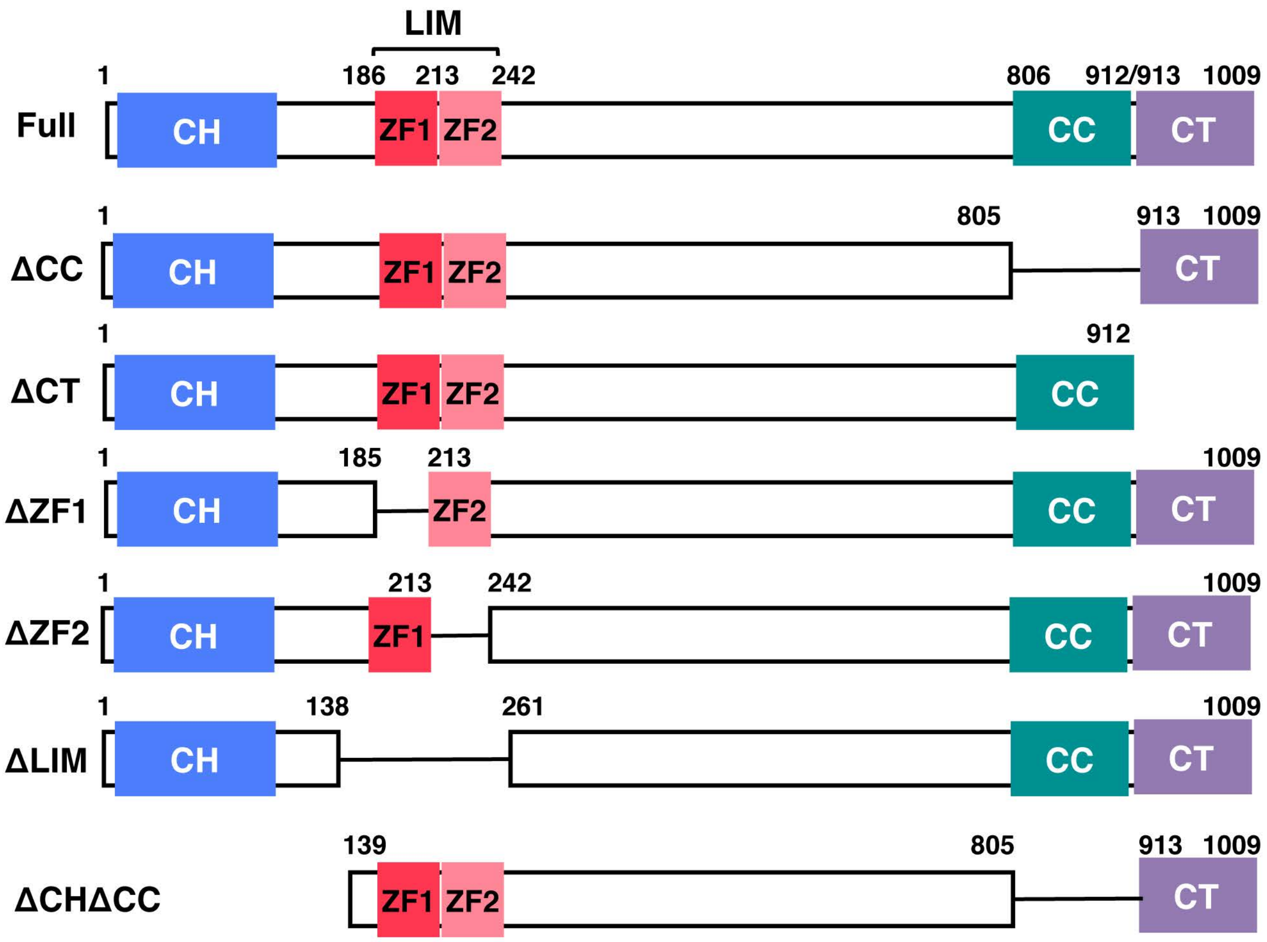

Supplementary Fig. S1

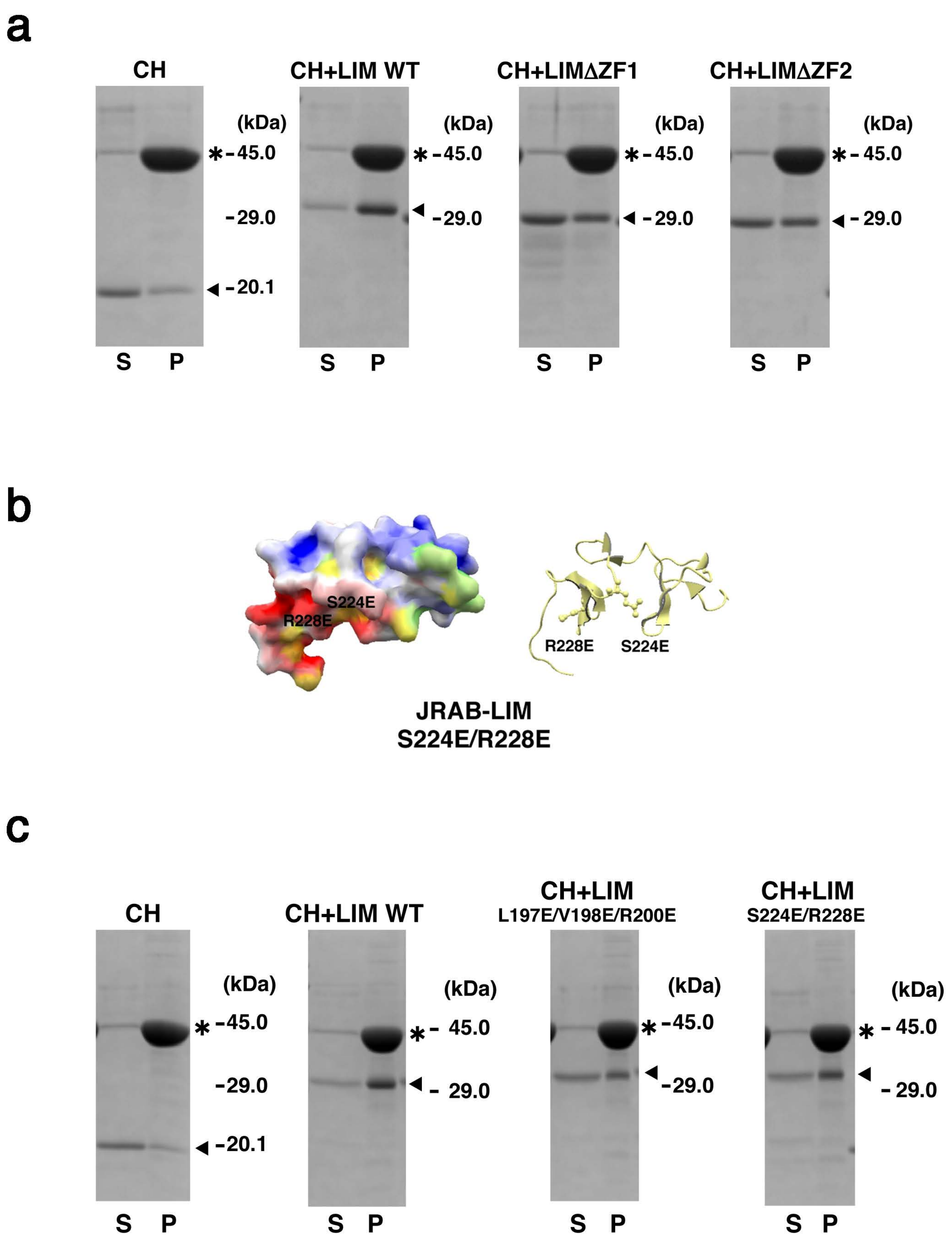

Supplementary Fig. S2

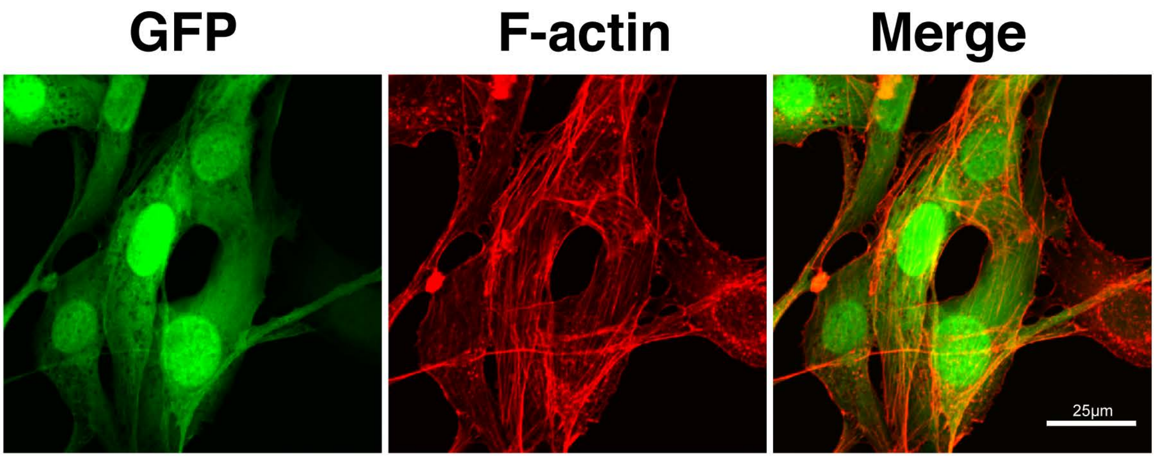

Supplementary Fig. S3

a

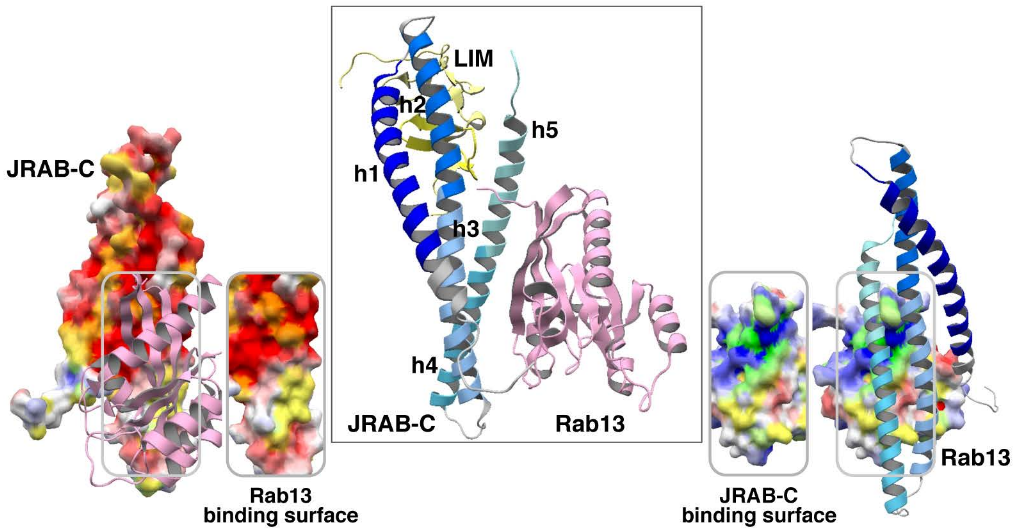

b

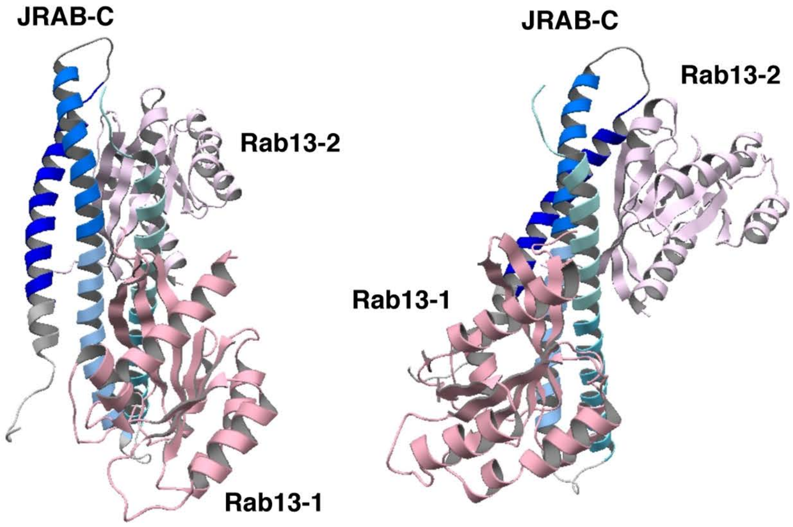

Supplementary Fig. S4

```
          920       930       940       950       960       970
MICAL-1   KEEEMKRFCKAQTIQRRLNEIEAALRELEAEGVKLELALRRQSSSPEQQKKLWVGQLLQL
                     ...::.:..::..: .::  ::.::  :: ......... .   ....:
JRAB-C            IPQEELQRQLQDIESQLDALELRGVELEKRLRAAEGDASEDSLMV--DWFRL
                         850       860       870       880         890

          980       990      1000      1010      1020      1030
MICAL-1   VDKKNSLVAEEAELMITVQELNLEEKQWQLDQELRGYMNREENLKTAADRQAEDQVLRKL
          ...:. :.  :.::: . .. .:::.:..:. ::: .:...:.::...::: :...:...
JRAB-C    IHEKQLLLRLESELMYKSKDQRLEEQQLDLQGELRRLMDKPEGLKSPQDRQREQELLSQY
                 900       910       920       930       940       950

         1040      1050       1060
MICAL-1   VDLVNQRDALIRFQEERRLSELALGTGAQG
          :. ::.:.... : .: ::.:
JRAB-C    VNTVNDRSDIVDFLDEDRLREQEEDQMLENMIQNLGLQRKKSKSFLSKIWSSKSKSGQA
                 960       970       980       990      1000
```

Supplementary Fig. S5

Figure 2a

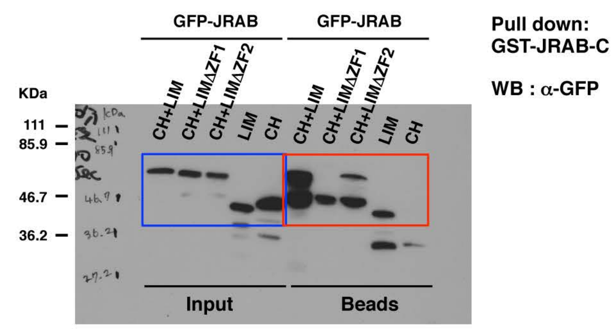

Figure 2b

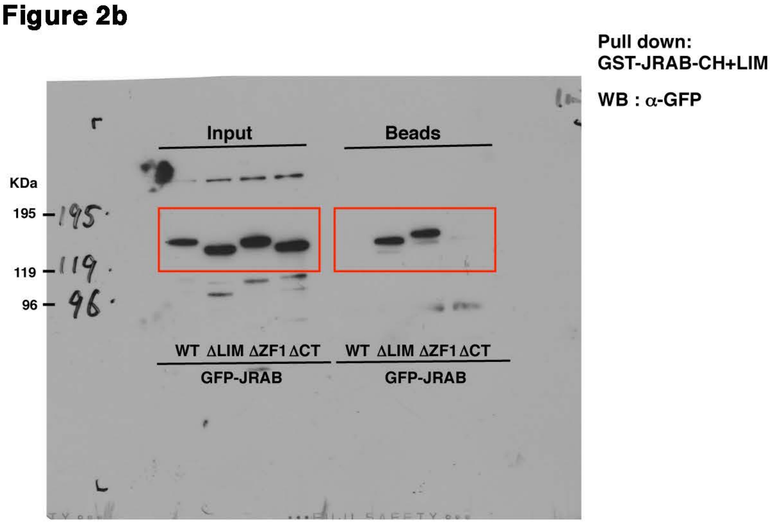

Figure 3d

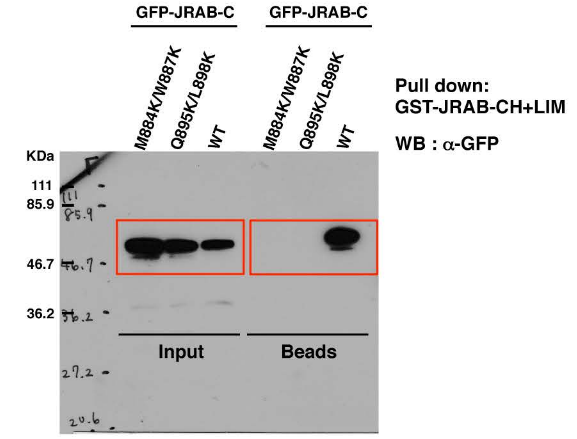

Figure 3e

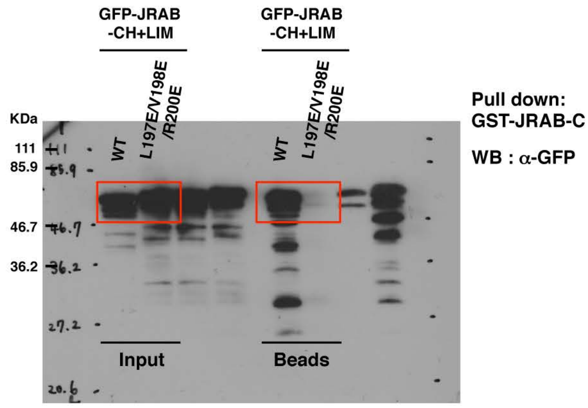

Figure 3f

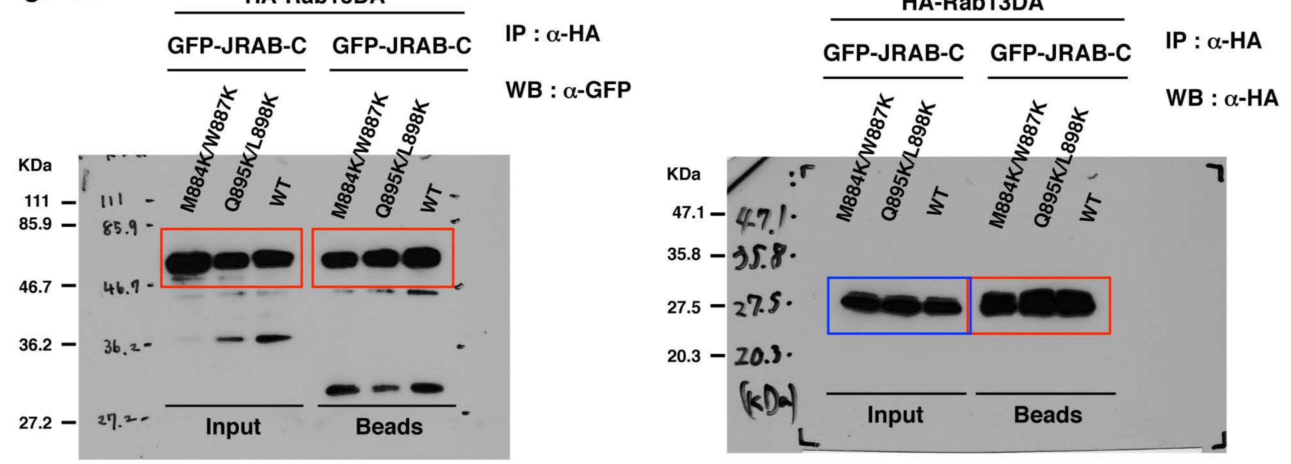

Figure 3g

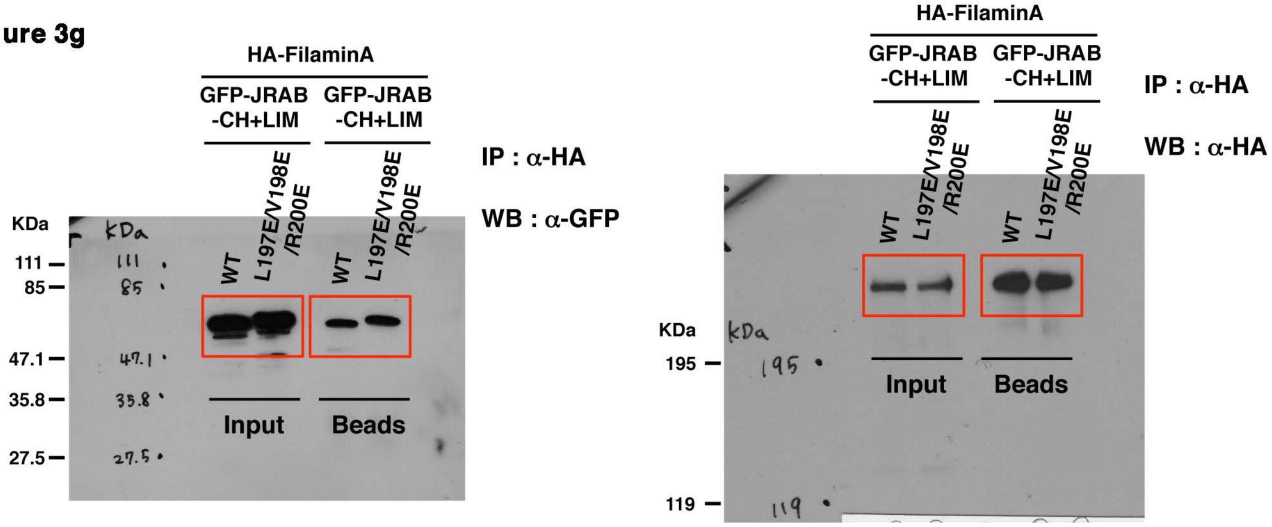

Supplementary Fig. S6

Figure 4d

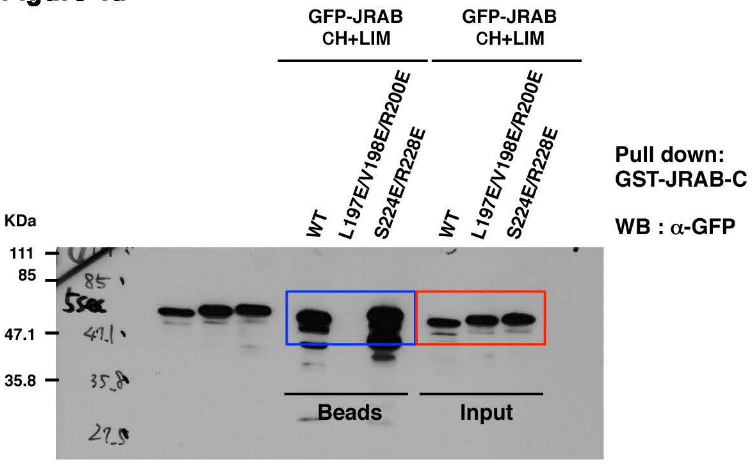

Figure 5b

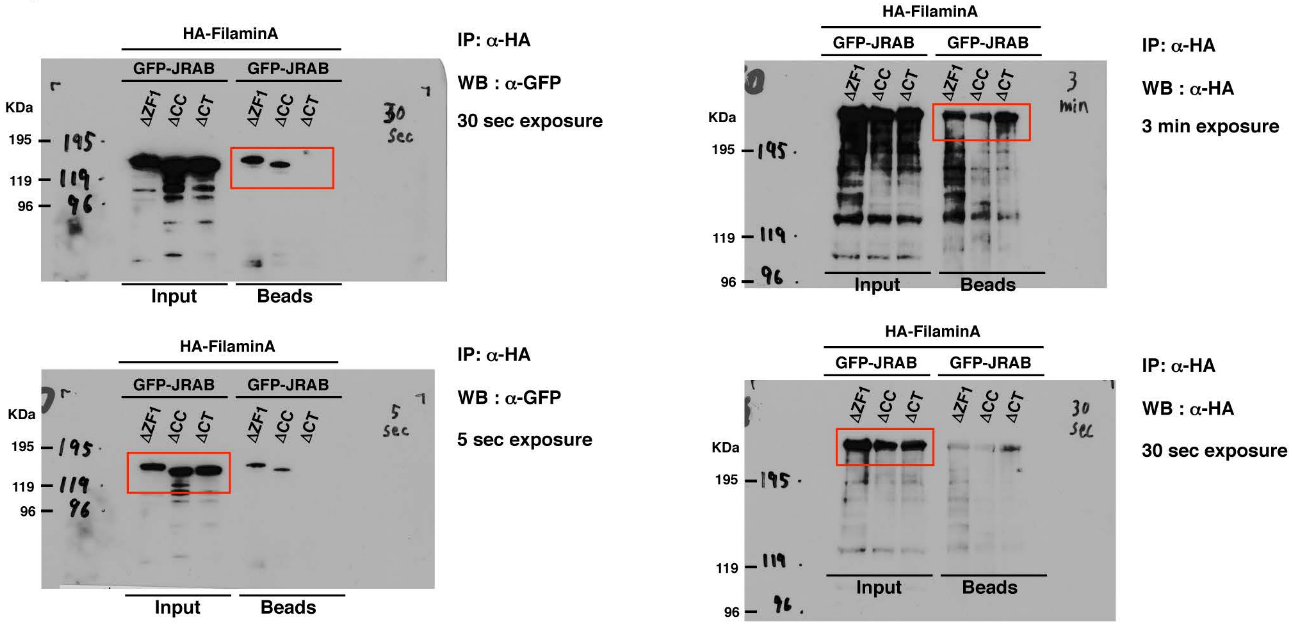

Supplementary Figure S2a

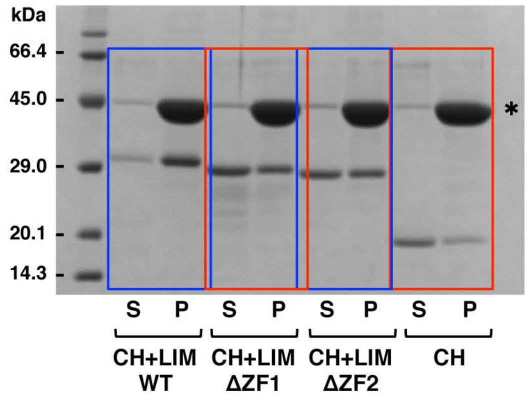

Supplementary Figure S2c

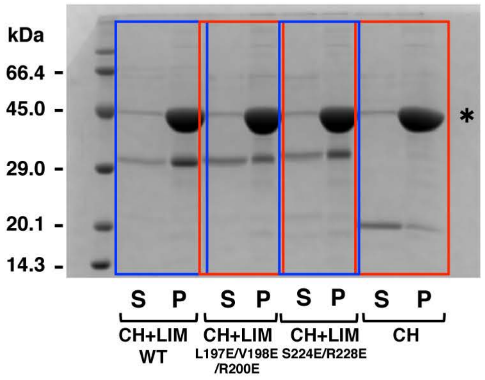

Supplementary Fig. S7
